# A loose endosperm structure of wheat seed produced under low nitrogen level promotes early germination by accelerating water uptake

**DOI:** 10.1038/s41598-017-03333-4

**Published:** 2017-06-08

**Authors:** Daxing Wen, Haicheng Xu, Liuyong Xie, Mingrong He, Hongcun Hou, Chunqing Zhang

**Affiliations:** State Key Laboratory of Crop Biology, Agronomy College, Shandong Agricultural University, Tai’an, Shandong Province 271018 P.R. China

## Abstract

Water uptake is the fundamental requirement for the initiation and completion of seed germination that is a vital phase in the life cycle of seed plants. We found that seeds produced under four nitrogen levels showed significantly different germination speed. The objective of this study was to study the mechanism of rapid seed germination and explore which pathways and genes play critical roles in radicle protrusion. Anatomical data revealed that seed protein content affected endosperm structure of seeds. Moreover, scanning electron microscope maps showed that faster germinated seeds had a looser endosperm structure compared with other seeds. Subsequently, high throughout RNA-seq data were used to compare the transcriptomes of imbibed seeds with different germination speed. Gene ontology (GO) term enrichment analysis revealed that cell wall metabolism related genes significantly up-regulated in faster germinated seeds. In these genes, the top four were chitinase that had about fourfold higher expression in faster germinated seeds. Kyoto Encyclopedia of Genes and Genomes (KEGG) enrichment analysis showed that faster germinated seeds had enhanced expression in glutathione metabolism. By combining these results, we propose a model for nitrogen fertilizer affects germination speed of wheat seed, which provide new insights into seed germination.

## Introduction

Germination is a vital phase in the life cycle of seed plants. Most seeds acquire the ability to germinate during development. Germination starts with the uptake of water by mature dry seed and finishes with radicle protrusion. Seed germination is a complex process influenced by many factors, such as genotype, environmental conditions^1, 2^. Some species of seeds are dormant at the end of development, which is an adaptive strategy for seeds to survive under adverse environmental conditions. However, in view of agricultural production, it creates an obstacle for rapid germination and seedling growth^3^. Dormancy can be overcame by physical or chemical treatments, such as water immersion, chemical and mechanical scarifcation. For example, plasma treatment can remarkablely change the hydrophilicity, water uptake and percentage of seed germination in *E*. *velutina* ^4^.

Transcriptomic and proteomic studies reveal many metabolism pathway in response to seed germination and seedling establishment, such as glycolytic pathway, defense response and lipid metabolism^3, 5–7^. Moreover, phytohormones also play an important role in seed germination and seedling establishment. In seed dormancy and germination, ABA/GA balance constitutes the central node in the interactions of diverse hormonal signals, and recent findings demonstrate that auxin is also critical for inducing and maintaining seed dormancy^8^. Arabidopsis WRKY6 transcription factor directly down-regulates *RAV1* expression in ABA signaling, and subsequently RAV1 directly represses the expression of *ABI3*, *ABI4* and *ABI5* for mediating seed germination and early seedling establishment^9^. A proteomic study indicates that GAs occurring is coinciding with or very close to radicle emergence in seeds of the GA deficient Arabidopsis *ga1* mutant^2, 10^.

The movement of water into dry mature seeds is a critical step in germination^11^. Previous researches have reported many methods to investigate water uptake during seed germination. The water penetration into a rice grain during soaking is monitored by using three-dimensional gradient echo magnetic resonance imaging^12^. Moreover, using Magnetic Resonance Micro-Imaging (MRMI) study the movement of water into harvest-ripe seeds of dormant and non-dormant genotypes in wheat^11^.

Seeds biosynthesize and deposite carbohydrates, oils and proteins during seed development, which are critical for seed germination and seedling establishment^3^. However, very few studies have investigated the mechanism underlying nitrogen fertilizer affecting seed germination. In this study, we found that seeds produced under four nitrogen levels showed different germination speed. Storage proteins not only supply nutrients for seed germination and seedling establishment but also influence on water uptake by changing the endosperm structure.

Previous studies have revealed that seed compartments (testa, endosperm, and embryo) control germination, and constructed a meaningful coexpression networks during germination by the potential of a high-resolution data set^13^. However, which pathways and genes play critical roles in the radicle protrusion of faster germinated seeds are still unknown. In this study, we used RNA-seq data to analyze the difference in transcriptome level between faster germinated seeds and slower germinated seeds at early stage of germination. Taken together, we propose a model for nitrogen fertilizer affects seed germination speed in wheat, which provides new insights into water uptake and seed germination.

## Results

### Comparison of germination speed of seeds produced under four nitrogen levels

**Figure 1:**
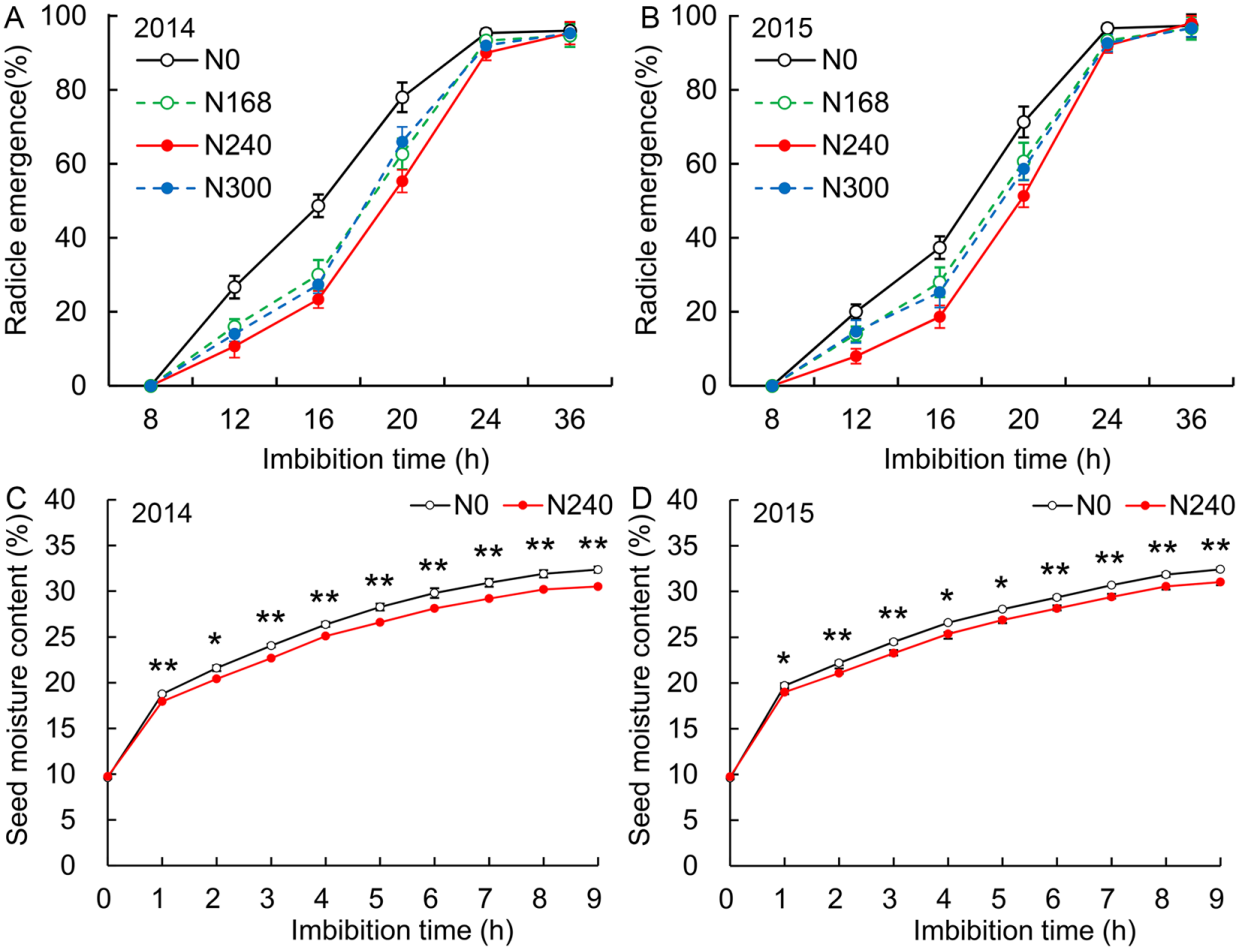
Radicle emergence and seed moisture content under four nitrogen levels. Four nitrogen levels: N0 (0 kg/ha), N168 (168 kg/ha), N240 (240 kg/ha, the usual nitrogen fertilizer level for winter wheat production in the North China Plain) and N300 (300 kg/ha). (**A**,**B**) Radicle emergence of seeds in 2014 and 2015. (**C**,**D**) Seed moisture content in 2014 and 2015. Error bars represent the standard deviation for three field plot replicates and each field plot replicate includes at least three technical replicates. Asterisks denote a significant difference according to unpaired Student’s t test (*p ≤ 0.05; **p ≤ 0.01).

Seeds produced under four nitrogen fertilizer levels, that is, N0 (0 kg/ha), N168 (168 kg/ha), N240 (240 kg/ha, the usual nitrogen fertilizer level for winter wheat production in the North China Plain) and N300 (300 kg/ha). N168 (168 kg/ha) is 30% less than N240. N300 (300 kg/ha) is almost 30% more than N240, which is usually high nitrogen fertilizer level used by local peasants. We analyzed seed germination and a seed was considered radicle emergence when protrusion of the radicle from the seed coat was observed. Seeds under N0 and N240 showed the highest and lowest radicle emergence from 12 to 24 h after imbibition (HAI) in two years, respectively (Fig. 1A and B). Seeds under N168 and N300 had slightly higher radicle emergence than N240 seeds, and all of seeds under four nitrogen levels showed at least 95% at 36 HAI. These observations indicated that N0 seeds germinated more rapidly than seeds under N168, N240 and N300. We know that seed can not germinate until seed moisture content reaching a certain degree. Thus, we analyzed seed moisture content in more details. N0 seeds showed significantly higher seed moisture content from 1 to 9 HAI compared with N240 seeds (Fig. 1C and D). For example, seed moisture content reaching 30% in 2014 needed about 6 and 8 h in N0 seeds and N240 seeds, respectively. The results confirmed that N0 seeds germinated more rapidly than N240 seeds.

### A loose endosperm structure accelerates seed germination

**Figure 2:**
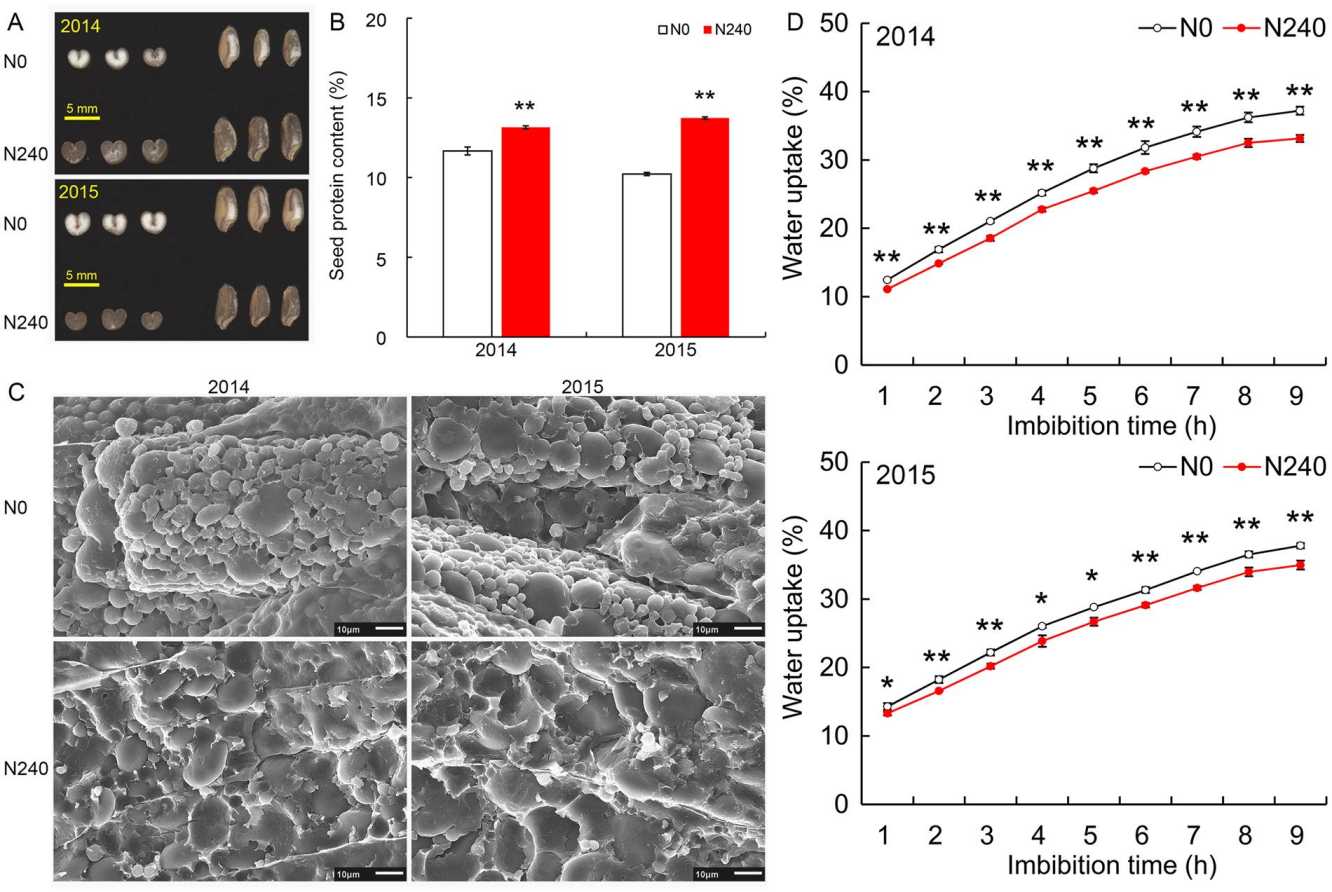
Effects of endosperm structure of seeds on water uptake. (**A**) Transverse section and longitudinal section of N0 seeds and N240 seeds. (**B**) Protein content of N0 seeds and N240 seeds. (**C**) Scanning electron microscope maps enlarging 1000 × endosperm structure of N0 seeds and N240 seeds. (**D**) Water uptake of seeds. Error bars represent the standard deviation for three replicates. Asterisks denote a significant difference according to unpaired Student’s t test (*p ≤ 0.05; **p ≤ 0.01).

To further study the reason why N0 seeds showed significantly higher germination speed and seed moisture content compared with N240 seeds, we analyzed seed size, endosperm structure and water uptake. It was fluctuant in 1000-grain weight among four nitrogen fertilizer levels in two years (Supplementary Table S1). Seeds under N168 and N0 had the biggest and the smallest 1000-grain weight in 2014, respectively. Due to severe dry and hot wind on June 6th, 2015, grain filling was completed ahead of time. Rainfall recorded during the period of wheat growth (October to June) in 2014–2015 was higher than that in 2013–2014, but June rainfall was lower in 2015 than in 2014 (Supplementary Fig. S1A and B). June mean temperature was higher in 2015 than in 2014. Daily maximum temperature and daily mean temperature of one week before harvest were also higher in 2015 than in 2014 (Supplementary Fig. S1C and D). N0 and N300 had the biggest and the smallest 1000-grain weight at harvest, respectively. Therefore, 1000-grain weight could not reflect germination speed. Moreover, we surveyed seed length and width under four nitrogen fertilizer levels in two years (Supplementary Table S1). N300 in 2015 were not shown in Supplementary Table S1 because it appeared large scale plant lodging and shrivelled kernel. Seed width in 2014 were different from it in 2015, which was due to severe dry and hot wind affected grain filling in 2015. Seed length and width affect 1000-grain weight. Subsequently, we used microscopic analyses to investigate the internal structure of seeds. For transverse section and longitudinal section, we observed that N0 seeds and N240 seeds were powder seeds and cutin seeds in two years, respectively (Fig. 2A). N240 seeds had significantly higher seed protein content than N0 seeds in two years (Fig. 2B). The endosperm structure of N0 seeds were more interspace and starch granule than N240 seeds in scanning electron microscope maps in two years (Fig. 2C). N0 seeds showed remarkably higher water uptake from 1 to 9 HAI compared with N240 seeds, indicating that a loose endosperm structure accelerates water uptake (Fig. 2D). Starch is the prime storage reserve in wheat seeds and provides the main source of carbohydrate for germination and seedling establishment. Among hydrolases, α-amylase plays a central role in starch decomposition. The activities of α-amylase under N0 seeds were significantly higher than that under N240 seeds at both 8 and 16 HAI in two years (Supplementary Fig. S2), suggesting that N0 seeds germinated more rapidly than N240 seeds. These results further confirmed that N0 seeds absorbed water more rapidly compared with N240 seeds, thereby N0 seeds germinated more rapidly than N240 seeds.

### Comparison of gene expression at early stage of seed germination

To explore pathways and genes that affect seed germination speed and control radicle protrusion in wheat, we used RNA-seq to analysis the transcriptomes of 8 h imbibed seeds under N0 and N240 in 2015. High-throughput RNA-seq generated 41 to 51 million reads for each sample, and three biological replicates were performed for each treatment (Supplementary Table S2). About 40 to 49 million clean reads were remained after removing adapter sequences and low quality regions, and then about 29 to 36 million clean reads were mapped to the wheat genome. Of the clean reads in each library, 10.55–11.52% were mapped to multiple locations (Multiple mapped reads), and 62.56–62.96% were mapped to single locations in the reference sequence (Uniquely mapped reads). Using false discovery rate (FDR) < 0.05 as the significance cutoffs, we used the R package edgeR to identify differentially expressed genes (DEGs). We found that 415 genes were significantly down-regulated and 151 genes were significantly up-regulated in 8 h imbibed seeds under N240 (B8) compared with those under N0 (A8).

### Validation of RNA-Seq data by qRT-PCR

**Figure 3:**
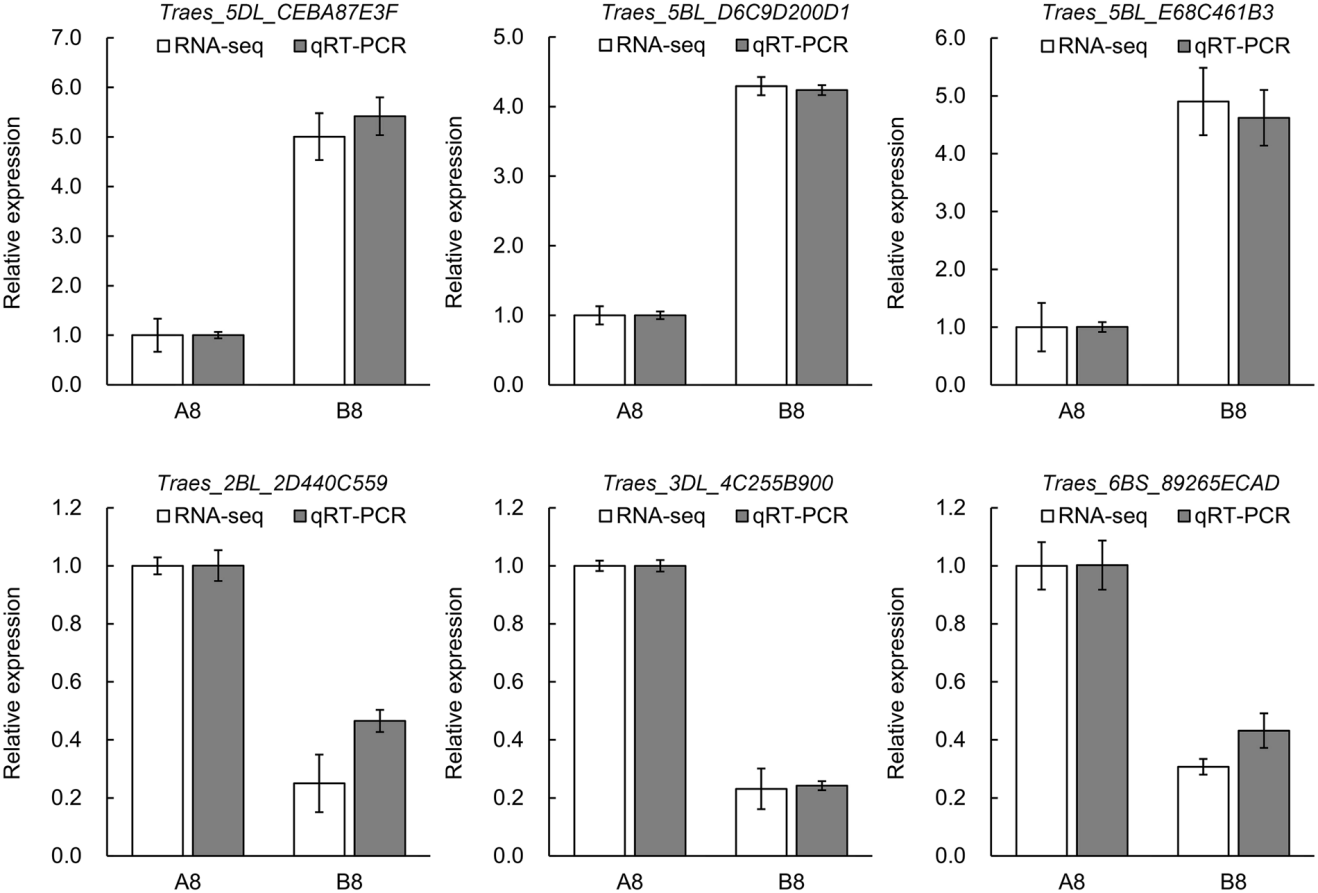
Validation of differentially expressed genes by qRT-PCR. A8: N0 seeds at 8 HAI. B8: N240 seeds at 8 HAI. The relative expression level of each gene was expressed as the fold change between N0 and N240 in the RNA-Seq data (white bar) and qRT-PCR data (gray bar). The wheat Actin gene was used as an internal control to normalize the expression data. Error bars represent the standard deviation for three replicates.

To validate the DEGs identified by RNA-Seq, we performed quantitative real time PCR (qRT-PCR) assays. Among the six randomly selected DEGs, three genes displayed up-regulation and three genes showed down-regulation in B8. As shown in Fig. 3, all the six genes showed the similar expression patterns in the qRT-PCR assays as their transcript abundance changes identified by RNA-seq, indicating that the RNA-seq data were credible.

### Cell wall metabolism related genes show enhanced expression in faster germinated seeds

**Figure 4:**
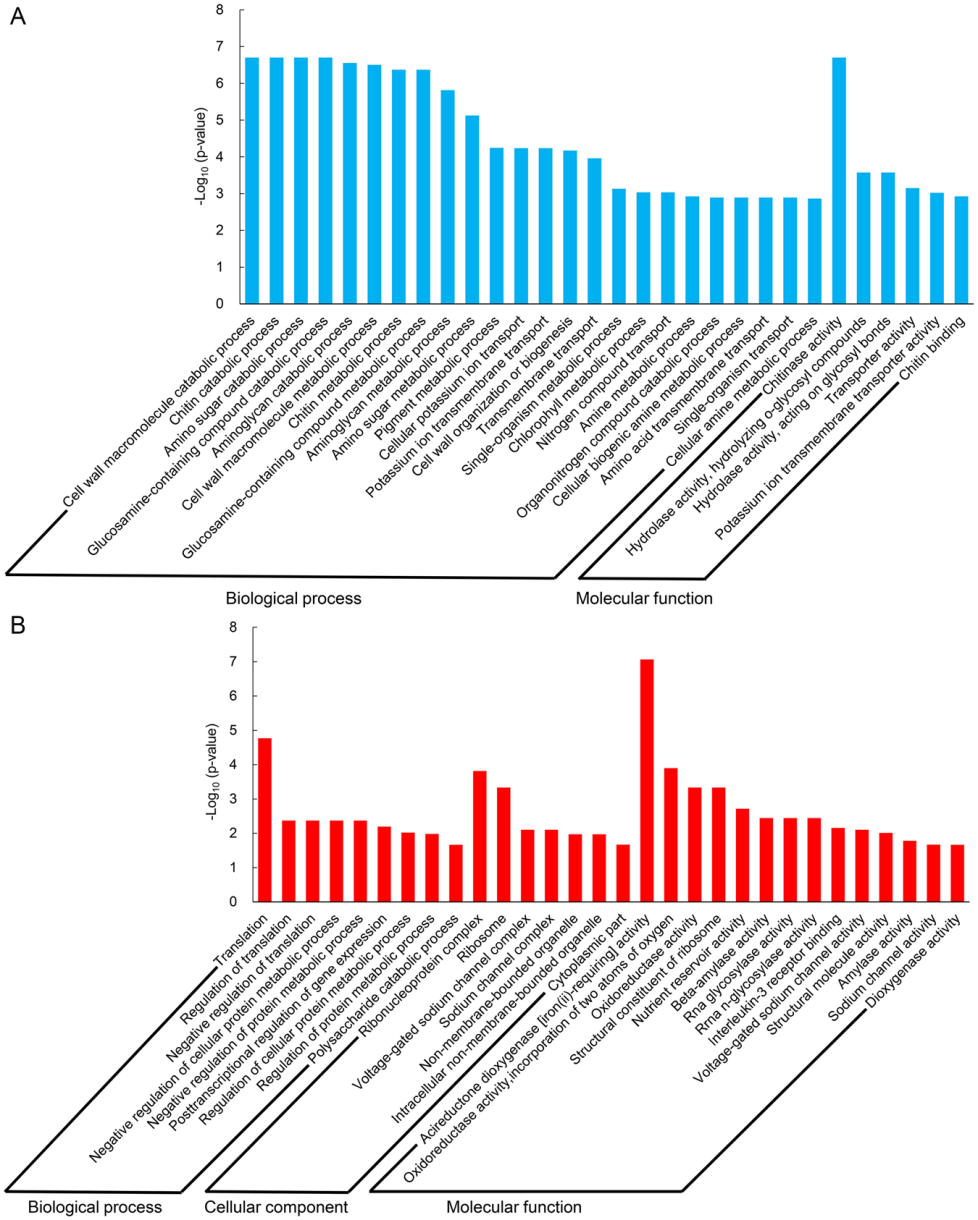
Significantly enriched Gene Ontology (GO) terms (p < 0.05) in B8 VS A8. A8: N0 seeds at 8 HAI. B8: N240 seeds at 8 HAI. (**A**) GO terms for the down regulated genes. (**B**) GO terms for the up regulated genes. GO terms were sorted based on p-values.

To further understand the function of these DEGs, gene ontology (GO) term enrichment analysis (p < 0.05) was performed. The top 30 GO terms involved in down-regulated and up-regulated genes are shown in Fig. 4A and B, respectively. For genes that were down regulated in 8 h imbibed seeds of N240 compared with those of N0 (B8 VS A8), the most significantly enriched GO terms were cell wall macromolecule catabolic process (GO: 0016998, p = 1.99E-7) in the biological process group and chitinase activity (GO: 0004568, p = 1.99E-7) in the molecular function group (Fig. 4A), respectively. The two GO terms are related to breakdown of macromolecules that form part of a cell wall. Moreover, Cell wall organization or biogenesis (GO: 0071554, p = 3.13E-7), chitin catabolic process (GO: 0006032, p = 1.99E-7), chitin metabolic process (GO: 0006030, p = 4.26E-7) and chitin binding (GO: 0008061, p = 1.18E-3) were also significantly enriched in B8 VS A8. There were 0% and about 15% radicle emergence at 8 and 12 HAI, respectively (Fig. 1A and B). Many cell wall metabolism related GO terms significantly enriched in B8 VS A8, indicating that 8 h imbibed seeds prepared for radicle protrusion. Thirty cell wall metabolism related genes that had lower expression in B8 were listed in Table 1. The top four genes were chitinase that had about four-fold lower expression in B8 VS A8. Besides many chitinase related genes, pectinesterase (*Traes_1AL_1C66B5A24* and *Traes_1BL_5E924707C*), beta-glucosidase (*Traes_3AL_4DB83ED86* and *Traes_3DL_0CA0B746D*), xyloglucan endotransglucosylase/hydrolase protein 13 (*Traes_2BL_232323148*), cell number regulator (*Traes_5DL_8B9E316C1* and *Traes_4AL_B98797B11*) and expansin (*Traes_1BL_8E3D5E5DF* and *Traes_1DL_8E0395E20*) related genes were down-regulated in B8 VS A8. Therefore, these results illustrated that N0 seeds had higher cell wall metabolism level involved in seed germination and radicle protrusion than N240 seeds at 8 HAI, thereby confirming that N0 seeds germinated more rapidly than N240 seeds.

**Table 1:** List of selected genes about cell wall metabolism that were differentially expressed in B8 VS A8.

Gene ID	Gene Annotation	log2FoldChange	p-value
Traes_1BL_081C896A0	Chitinase 2	−2.0215	2.69E-04
Traes_2BL_2D440C559	Chitinase 5	−1.9757	7.70E-03
Traes_2AL_6162A036E	Chitinase 5	−1.9254	9.72E-05
Traes_1DL_95936DC50	Chitinase 8	−1.7274	1.07E-03
Traes_1AL_1C66B5A24	Probable pectinesterase/pectinesterase inhibitor 41	−1.4998	1.56E-03
Traes_4DL_702BA9725	L-type lectin-domain containing receptor kinase IX.1	−1.4197	3.65E-02
Traes_3AL_4DB83ED86	Glucan endo-1,3-beta-glucosidase GII	−1.1096	2.65E-04
Traes_5DL_8B9E316C1	Cell number regulator 10	−1.0763	9.64E-03
Traes_2AL_AE6CC81B2	LRR receptor-like serine/threonine-protein kinase	−1.0483	2.66E-02
Traes_4BL_2F1CAFF7E	L-type lectin-domain containing receptor kinase IX.1	−1.0007	3.91E-02
Traes_4AL_B98797B11	Cell number regulator 10	−0.9413	5.47E-04
Traes_3DL_0CA0B746D	Glucan endo-1,3-beta-glucosidase GI	−0.9384	4.77E-02
Traes_2BL_232323148	Putative xyloglucan endotransglucosylase/hydrolase protein 13	−0.9363	3.24E-09
Traes_1BL_8E3D5E5DF	Expansin-B3	−0.7622	3.59E-12
Traes_1BL_5E924707C	Pectinesterase 31	−0.6960	2.20E-02
TRAES3BF280800020CFD_g	Basic endochitinase A	−0.6840	2.70E-02
Traes_1AL_E96C0662D	Chitinase 8	−0.6768	2.11E-02
Traes_5BL_2F730509F	Endoglucanase 22	−0.6141	3.53E-02
Traes_2AS_CE5E5F0A1	Probable LRR receptor-like serine/threonine-protein kinase	−0.6100	9.15E-03
Traes_1DL_8E0395E20	Expansin-B2	−0.5875	5.47E-04
Traes_2DL_C141AAB8D	Cytokinin dehydrogenase 8	−0.5600	1.14E-03
Traes_1AL_DDC13A76D	Expansin-B3	−0.5525	3.27E-06
Traes_2BL_E366A5226	Chitinase 4	−0.5247	5.87E-04
Traes_5BL_58CAE6F7B	Beta-glucosidase 31	−0.5074	4.47E-04
Traes_1DL_9F02FFABA	Beta-glucosidase 22	−0.4766	1.19E-02
Traes_7AL_E4FD8421A	26 kDa endochitinase 1	−0.4556	1.88E-04
Traes_4AL_13B97C7E5	Glucan endo-1,3-beta-glucosidase	−0.4035	2.10E-02
Traes_2AL_C987E68952	Chitinase 5	−0.3936	8.43E-03
Traes_5AL_133FEF770	Beta-glucosidase 31	−0.3796	3.96E-03
Traes_2AL_72C5A8EE7	Chitinase 5	−0.3299	3.13E-02

### Protein metabolism related genes may be implicated in seed protein content

For the up regulated genes in B8 VS A8, the most remarkably enriched GO terms were translation (GO: 0006412, p = 1.70E-5) in the biological process group, ribonucleoprotein complex (GO: 0030529, p = 1.52E-4) in the cellular component group, and acireductone dioxygenase [iron (II)-requiring] activity (GO: 0010309, p = 8.55E-8) in the molecular function group, respectively (Fig. 4B). The results showed that protein metabolism in B8 was higher than that in A8, which might be due to higher protein content in N240 seeds. Twenty protein metabolism related genes that had higher expression in B8 were listed in Supplementary Table S3. Protein synthesis inhibitor (*Traes_5DL_CEBA87E3F*, *Traes_5BL_D6C9D200D1* and *Traes_5DL_DF4EA7BE9*), protein phosphatase (*Traes_5BL_BF328BC61*), 60 S ribosomal protein (*Traes_7BS_5ECCA936B* and *Traes_6DL_03A05A5D7*) and 40 S ribosomal protein (*Traes_1AS_CDFADEA22* and *Traes_2BS_9FC5D6F3A*) showed elevated transcription level in B8.

### Glutathione metabolism plays an important role in faster germinated seeds

**Figure 5:**
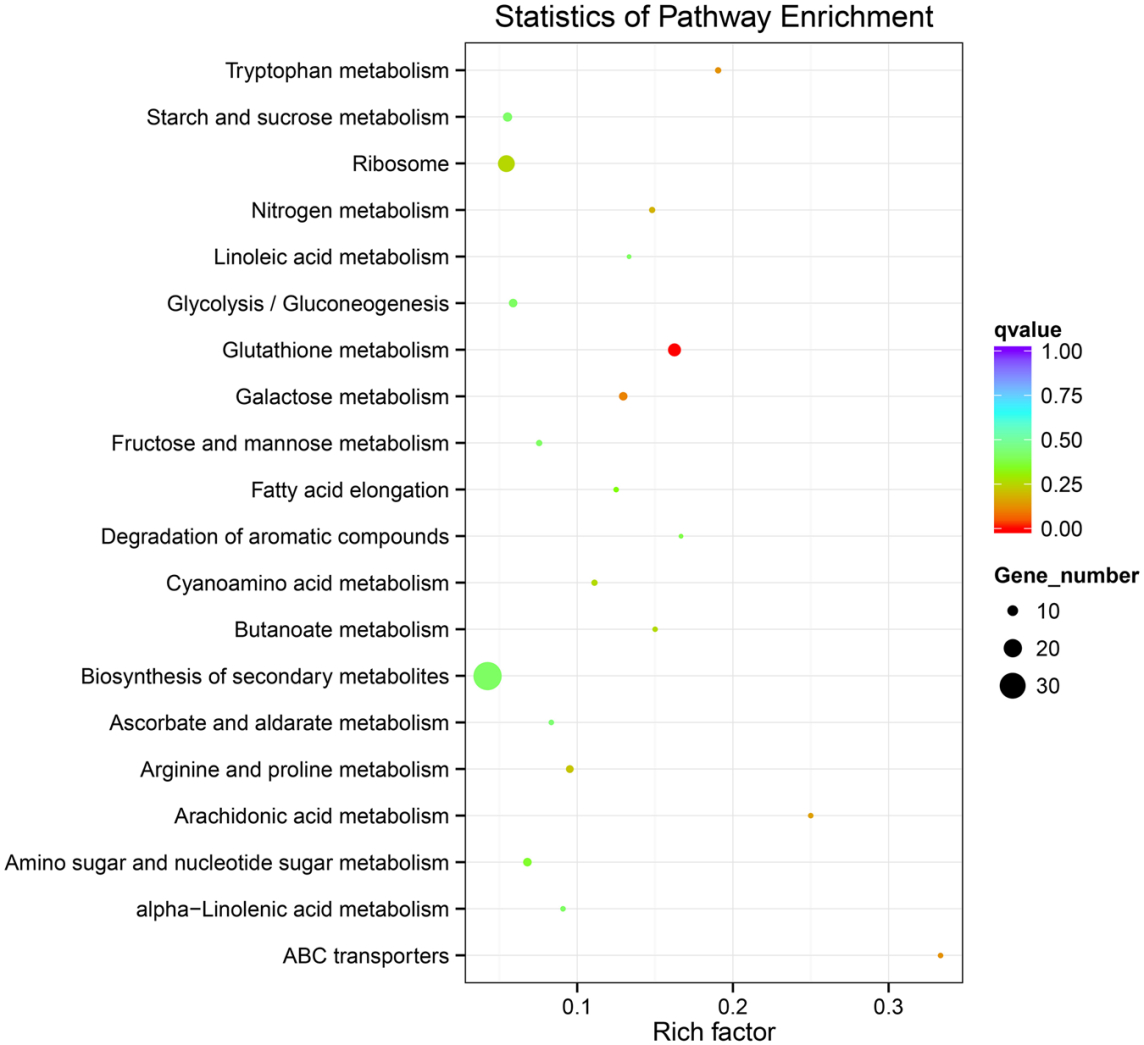
Significantly enriched Kyoto Encyclopedia of Genes and Genomes (KEGG) pathways (p < 0.05) in B8 VS A8. A8: N0 seeds at 8 HAI. B8: N240 seeds at 8 HAI.

To identify the pathways that are active in faster geminated seeds, we mapped all of the DEGs to reference pathways in the Kyoto Encyclopedia of Genes and Genomes (KEGG). Glutathione metabolism was the most significantly enriched pathway in B8 VS A8 (Fig. 5). Glutathione peroxidase [EC:1.11.1.9], glutathione S-transferase [EC:2.5.1.18] and ornithine decarboxylase [EC:4.1.1.17] were enriched in glutathione metabolism. All of the DEGs enriched in glutathione metabolism were down-regulated in B8 (Table 2). Glutathione metabolism is related to scavenge reactive oxygen species (ROS). Previous researches have revealed that seeds generate ROS during germination. The results indicated that faster germinated seeds had higher the ability of scavenging ROS than other seeds.

**Table 2:** List of DEGs enriched in glutathione metabolism pathway.

KO	EC	UniGenes	log2FoldChange	p-value
K00432	glutathione peroxidase [EC:1.11.1.9]	Traes_2DL_1827C450E	−0.4068	3.27E-03
		Traes_2AL_97834165 F	−0.3995	6.72E-03
		Traes_2BL_B51F2BBE0	−0.3811	3.98E-02
K00799	glutathione S-transferase [EC:2.5.1.18]	Traes_4AL_80727DCA6	−3.9649	2.24E-02
		Traes_1AL_CA2AFD745	−0.9900	2.57E-05
		TRAES3BF156400110CFD_g	−0.7465	3.17E-10
		Traes_3AS_F434A9F61	−0.6648	1.40E-02
		Traes_3AS_9165253EC	−0.6091	3.66E-02
		Traes_4AL_26567AFFC	−0.5858	1.76E-02
		Traes_4AS_36CB7931F	−0.5856	5.56E-04
		Traes_3DS_3CD0E7C53	−0.4499	4.04E-03
		Traes_4BS_615DE1514	−0.3430	1.49E-02
K01581	ornithine decarboxylase [EC:4.1.1.17]	Traes_5BL_82626DD6E	−1.0605	2.91E-02

## Discussion

The endosperm structure of N0 seeds was looser than N240 seeds, causing N0 seeds accelerating radicle protrusion. Interestingly, previous studies show that a loose structure in opaque part of maize seeds is conducive to moisture diffusion while a dense structure of vitreous part is not^14^. The plasma treatment have a positive effect on *E*. *velutina* seed germination, which is due to seed coat erosion and etched/eroded surfaces when seeds immersed in plasma are subjected to attack by oxygen radicals and are bombarded by ions^4^. Moreover, the pattern and speed of water penetration are determined by the morphological structure, crack formation and hardness distribution involved in the packing of the starch granules in rice seeds^12^. These data suggest that changes of seed moisture content are influenced by structure of seeds, which is consistent with our results. Moreover, big seeds show slower water uptake than small seeds. This is in agreement with our observations that the difference of germination speed in 2015 was smaller than that in 2014 (Fig. 1A and B).

Storage protein is important nutrition for seed germination and seedling growth^6, 15^. Moreover, the proteins involved in cell defense and rescue are associated with increased seed desiccation tolerance and vigor^3^. At the early stage of water uptake, seed germination begins with a resumption of maturation program through the translation of mRNA associated with storage protein. In the lag phase, a sequential translation of mRNA related with antioxidant mechanisms, cell detoxification, protein fate, energy, and amino acids metabolism occurs^16^. In this study, nitrogen fertilizer affected seed protein content, thereby influenced on water uptake and germination speed. Translation (GO: 0006412, p = 1.70E-5) and ribonucleoprotein complex (GO: 0030529, p = 1.52E-4) were significantly enriched in GO terms, which could be related to protein content.

Chitinase genes is the most abundant in cell wall metabolism that were differentially expressed in B8 VS A8. For example, *Traes_2BL_2D440C559* showed a four fold (log2FoldChange = −1.9757) lower expression in 8 h imbibed seeds under N240 compared with those under N0 (Table 1). In addition, qRT-PCR verified *Traes_2BL_2D440C559* had significantly lower expression in imbibed 8 h N240 seeds (Fig. 3). However, studies on chitinase mainly about cell wall catabolic and plant defense against pathogens. In recent years, more and more studies show that chitinase and chitinase-like (CTL) proteins have diverse functions on altering root system architecture^17^, affecting cellulose biosynthesis^18^ and the development of the gelatinous (G-type) cellulosic walls^19^. Therefore, we hypothesized that chitinase involved in radicle protrusion by regulating cell wall metabolism. Previous studies have shown that activation of cell division is important to promote successful seed germination and cell division within the radicle tip is important for their increased germination speed^1, 20^. This is in agreement with our observations that some cell division related genes showed down-regulation in 8 h imbibed N240 seeds (Table 1). For example, genes of cell number regulator 10 (*Traes_5DL_8B9E316C1* and *Traes_4AL_B98797B11*) and cytokinin dehydrogenase 8 (*Traes_2DL_C141AAB8D*) are related to cell division.

Ribosome-inactivating proteins (RIPs) are enzymes that inhibit protein synthesis after depurination of a specific adenine in rRNA^21^. The RIP family members are classified as type I RIPs that contain an RNA-N-glycosidase domain and type II RIPs that contain a lectin domain (B chain) in addition to the glycosidase domain (A chain). All of RIP genes were most highly expressed in the stages in which the endosperm was fully expanded in castor bean^21^. In this study, it might be that RIPs related genes (*Traes_5DL_CEBA87E3F*, *Traes_5BL_D6C9D200D1* and *Traes_5DL_DF4EA7BE9*) inhibited new protein synthesis in some parts of germinated seeds so that storage protein supplied nutrients for seedling growth (Supplementary Table S3).

Seeds of different species scavenge ROS caused by germination with many kinds of antioxidant system. In this study, KEGG enrichment analysis showed that only glutathione metabolism was significantly enriched in antioxidant system. Ascorbate peroxidases (APXs), one of the major antioxidants in plant cells, scavenge H_2_O_2_ and neutralize it by the glutathione–ascorbate cycle^3^. In this cycle, another three enzymes, monodehydroascorbate reductase (MDAR/MDHAR), glutathione-dependent dehydroascorbate reductase (DHAR), and glutathione reductase (GR) are involved in regenerating ascorbate back into the cellular antioxidant pool. In this study, N0 seeds had higher glutathione metabolism than N240 seeds at 8 HAI. Therefore, glutathione metabolism might play an important role in wheat seed germination.

**Figure 6:**
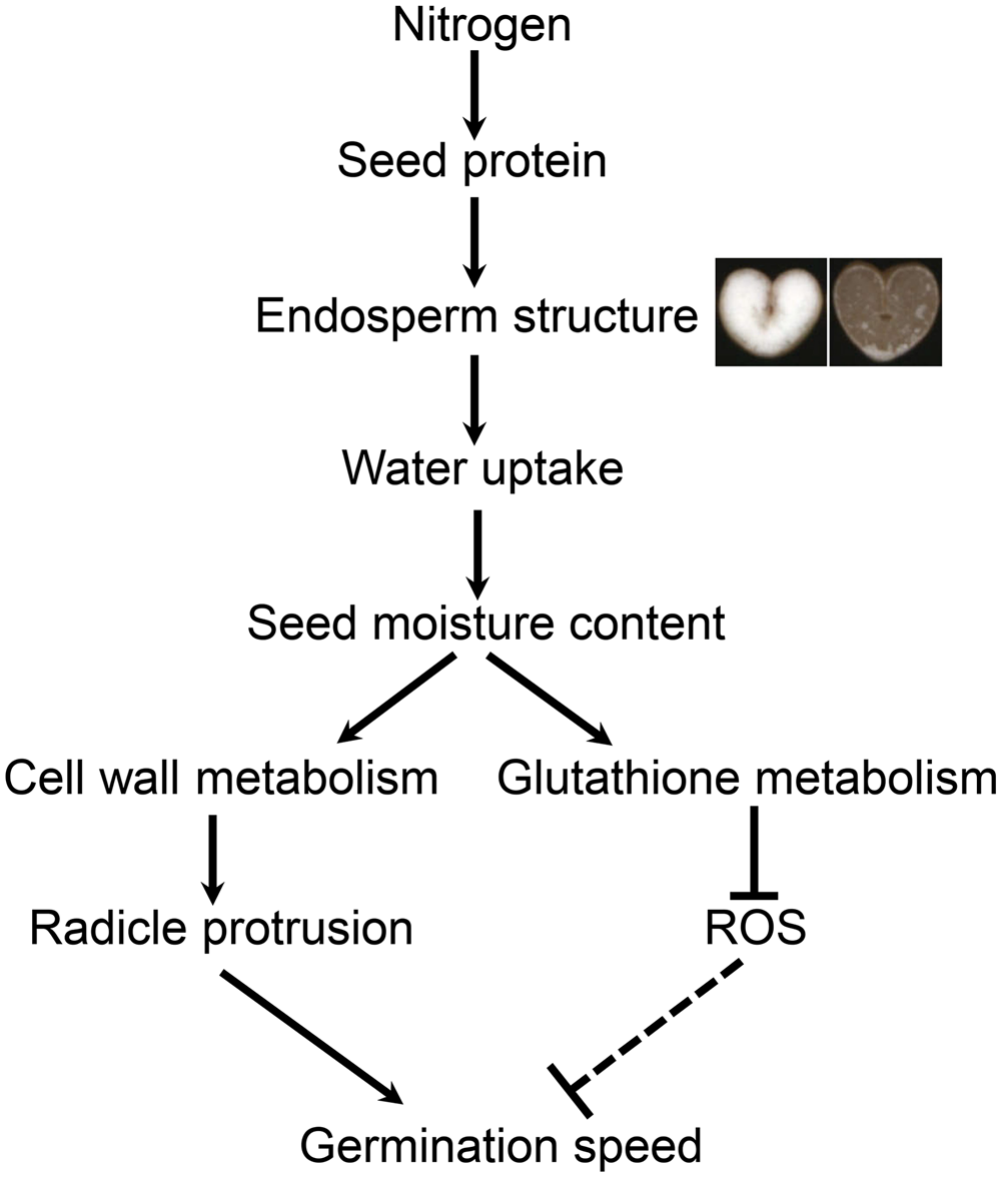
A hypothetical model for nitrogen affecting seed germination speed in wheat. Solid lines indicate the direct or definite regulation; dotted lines indicate the indirect or undetermined regulation.

In this study, we showed that a loose endosperm structure promotes seed germination by accelerating water uptake and cell wall metabolism play an important role in radicle protrusion. By combining these results, we propose a model for nitrogen fertilizer affected seed germination speed in wheat (Fig. 6). Nitrogen fertilizer affects seed protein content, resulting in the changes of endosperm structure and eventually influencing water uptake and seed moisture content. Enhanced expression of cell wall metabolism facilitates radicle protrusion thereby accelerating germination. Moreover, we speculate that ROS could slow down germination speed, which can be reduced by glutathione metabolism.

## Methods

### Materials

The winter wheat cultivar Tainong18 is a big-spike cultivar, which is planted widely in the North China Plain. It was grown in field trials in Dongwu Village (35°57′N and 117°3′E, Dawenkou Town, Tai’an City, Shandong Province, China) during 2013–2014 and 2014–2015. The area has a semi-humid continental temperate monsoon climate. The soil type is sandy loam.

### Experimental design

The trials were conducted using the randomized block design with three replications. The length and width of each field plot consisting of 12 lines was 40 m and 3 m, respectively. We set four nitrogen fertilizer levels, that is, N0 (0 kg/ha), N168 (168 kg/ha), N240 (240 kg/ha, the usual nitrogen fertilizer level for winter wheat production in the North China Plain) and N300 (300 kg/ha). We have conducted the trials seven times in continuous seven years and the near twice trials were used in this study. P (calcium superphosphate) and K (potassium chloride) were applied at rates of 120 kg/ha P_2_O_5_ and 75 kg/ha K_2_O, respectively. Base fertilizer was consisted of 40% nitrogen fertilizer, 100% phosphate fertilizer and 60% potash fertilizer, the residual 60% nitrogen fertilizer and 40% potash fertilizer as topdressing were used at jointing stage. Seeds were sowed on October 12th, 2013 and October 12th, 2014, respectively. The plant density was 450 plants/m^2^. Seeds were harvested on June 8th, 2014 and June 10th, 2015, respectively.

### Seed moisture content and 1000-grain weight

Seed moisture content was detected with DA7200 (Perten, Stockholm, Sweden). Three replications of 1000 seeds were randomly selected from each treatment for 1000-grain weight measurement.

### Seed size measurement

Three replications of 500 seeds each were randomly selected from each treatment. Seed length and width were measured with a Seed Analysis System (Wanshen, Hangzhou, China).

### Seed imbibition test

Three replications of 50 seeds each were randomly selected for seed imbibition test from 0 h to 9 h. Seed moisture content (SMC) and water uptake (WU) were calculated. SMC = (m_t_ − m_d_)/m_t_, WU = (m_t_ − m_0_)/m_d_, where m_t_ is seed weight at imbibition t h, m_d_ is seed dry weight and m_0_ is seed weight before imbibition.

### Internal structure of seeds

Internal structures of seeds were viewed by transverse section and longitudinal section. Scanning Electronic Microscopy was used to view enlarging 1000 × internal structure of seeds.

### Seed protein content

The seed nitrogen content was determined using the semi-micro Kjeldahl method, and the seed protein content was calculated by multiplying the seed nitrogen content by 5.7^22^.

### Statistical analysis

One-way analysis of variance, Duncan’s multiple tests and independent-samples T test were performed using SPSS 19.0 software (SPSS, Inc., Chicago, USA).

### RNA sequencing

Fifty imbibed seeds were pooled together as one biological sample for each treatment. Each treatment included three biological replicates. Samples were immediately frozen in liquid nitrogen and stored at −80 °C. Frozen samples were ground in a mortar with liquid nitrogen, and total RNA was extracted using the RNA extraction kit DP441 (Tiangen, Beijing, China). RNA was monitored on 1% agarose gels to avoid possible degradation and contamination, and then RNA purity was examined using the NanoPhotometer spectrophotometer (IMPLEN, CA, USA). RNA integrity was assessed using the RNA Nano 6000 Assay Kit of the Bioanalyzer 2100 system (Agilent Technologies, CA, USA). RNA concentration was measured using Qubit RNA Assay Kit in Qubit 2.0 Flurometer (Life Technologies, CA, USA).

Library construction and RNA sequencing were performed in Beijing Novogene Bioinformatics Technology Co., Ltd (Beijing, China). RNA-Seq librarys consruction were generated using NEBNext Ultra™ RNA Library Prep Kit for Illumina (NEB, USA) following manufacturer’s recommendations and index codes were added to attribute sequences to each sample. The libraries were sequenced on an Illumina Hiseq platform to generate 150 bp paired-end reads.

Raw data (raw reads) of fastq format were firstly processed to remove reads containing adapter, reads containing ploy-N and low quality reads. All the downstream analyses were based on the clean data with high quality. Clean reads were mapped to the wheat genome sequence (ftp://ftp.ensemblgenomes.org/pub/release-25/plants/fasta/triticum_aestivum/dna/) using TopHat v2.0.12^23^. The reads numbers mapped to each gene were counted using HTSeq v0.6.1, and then FPKM (expected number of Fragments Per Kilobase of transcript sequence per Millions base pairs sequenced) of each gene was calculated based on the length of the gene and reads count mapped to this gene. Differential expression analysis of two groups was performed using the DESeq R package (1.18.0)^24, 25^. The resulting p-values were adjusted using the Benjamini and Hochberg’s approach for controlling the false discovery rate. Genes with an adjusted p-value < 0.05 were assigned as differentially expressed.

Gene Ontology (GO) enrichment analysis of differentially expressed genes (DEGs) was performed using GOseq R package, in which gene length bias was corrected^26^. GO terms with corrected p-value < 0.05 were considered significantly enriched by DEGs. We used KOBAS software to test the statistical enrichment of DEGs in Kyoto Encyclopedia of Genes and Genomes (KEGG) pathways^27^.

### qRT-PCR

Primers for qRT-PCR were designed using the Primer 6 software and synthesized by Sangon Biotech (Shanghai, China). The gene specific primers are listed in Supplementary Table S4. cDNAs were reverse transcribed from total RNA using the PrimeScript RT reagent Kit (Takara, Dalian, China). Analyses of qRT-PCR were performed on an ABI Stepone plus Real-Time PCR System (Applied Biosystems, USA). Each qRT-PCR experiment was repeated three times. The wheat *Actin* gene was used as an internal control to normalize the expression data^28^. The relative expression level of genes was calculated using the 2^−ΔΔCt^ method and standard deviation was calculated between three biological replicates^29^.

### Alpha-amylase activity

Thirty germinated seeds were ground with moderate silica sand and 15 mL deionized water. The sample was placed in a 50 mL centrifuge tube and kept at room temperature for 20 min. It was then centrifuged at 3000 g for 10 min. The supernatant was diluted to 100 mL with deionized water, which was used to detect α-amylase activity by the 3, 5 - dinitrosalicylic acid method^30, 31^.

## Electronic supplementary material

Supplementary materials
