# Supplementary material for: A loose endosperm structure of wheat seed produced under low nitrogen level promotes early germination by accelerating water uptake
Source: Sci Rep. 2017 Jun 8;7:3116. doi: 10.1038/s41598-017-03333-4 (PMC5465190; doi:10.1038/s41598-017-03333-4)
Supplement: Supplementary file 1 — Supplementary materials [file 41598_2017_3333_MOESM1_ESM.pdf]

A loose endosperm structure of wheat seed produced under low nitrogen level promotes early germination by accelerating water uptake

Daxing Wen, Haicheng Xu, Liuyong Xie, Mingrong He, Hongcun Hou, Chunqing Zhang*

State Key Laboratory of Crop Biology, Agronomy College, Shandong Agricultural University, Tai'an, Shandong Province 271018, P. R. China

* Corresponding author: Chunqing Zhang

E-mail: cqzhang@sdau.edu.cn

Supplementary materials

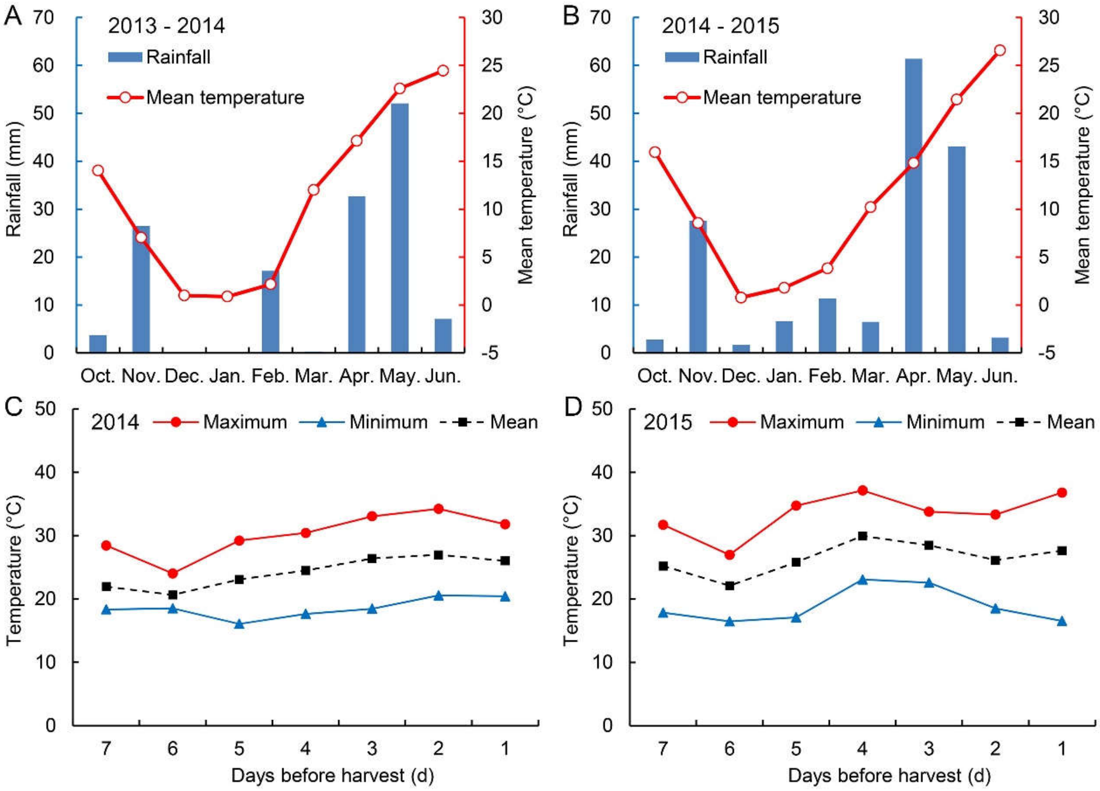

Supplementary Figure S1. Meteorological data recorded during the period of wheat growth (October to June). (A) and (B) Rainfall and mean temperature in 2013 – 2014 and 2014 – 2015. (C) and (D) Daily maximum temperature, daily minimum temperature and daily mean temperature of one week before harvest.

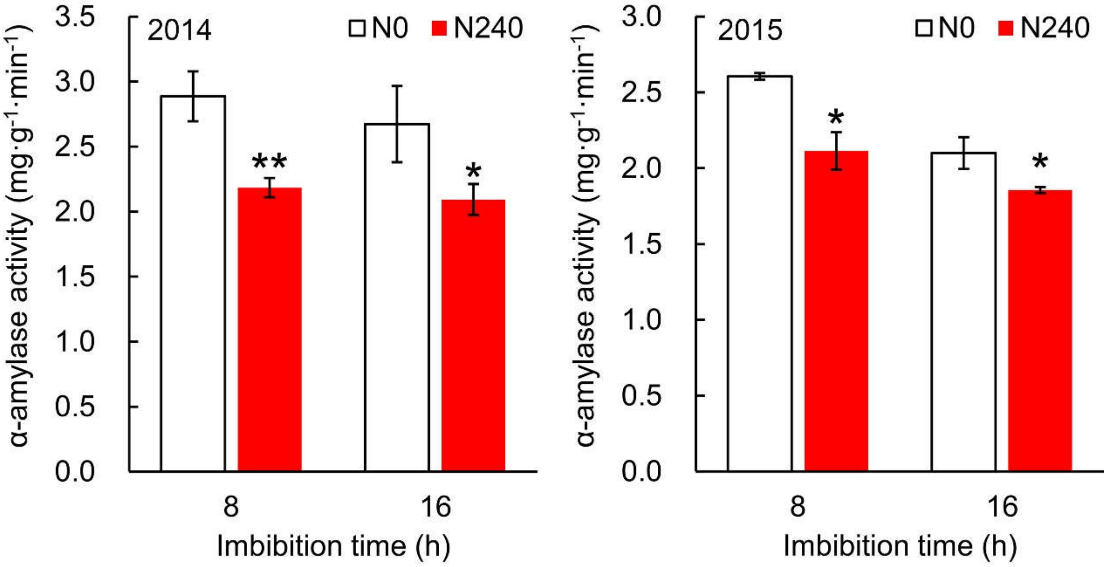

Supplementary Figure S2. Comparison of α-amylase activities between N0 seeds and N240 seeds at 8 and 16 HAI.

Supplementary Table S1. Effects of nitrogen fertilizer on seed size in two years

Year	Treatment	1000-grain weight (g)	Length (mm)	Width (mm)
2014	N0	34.2 ± 0.3 c	6.02 ± 0.12 b	3.32 ± 0.02 b
	N168	38.8 ± 0.4 a	6.35 ± 0.02 a	3.55 ± 0.05 a
	N240	38.3 ± 0.4 a	6.43 ± 0.01 a	3.57 ± 0.01 a
	N300	35.9 ± 0.5 b	6.32 ± 0.06 a	3.54 ± 0.01 a
2015	N0	37.0 ± 0.5 a	6.22 ± 0.01 b	3.51 ± 0.02 a
	N168	35.5 ± 0.2 b	6.46 ± 0.01 a	3.44 ± 0.03 a
	N240	35.2 ± 0.8 b	6.43 ± 0.03 a	3.41 ± 0.08 a
	N300	32.6 ± 0.4 c	NA	NA

The values represent the means ± standard deviation (n=3). Different letters indicated significant differences among means under different treatments (p value ≤ 0.05 by one-way ANOVA analysis).

Supplementary Table S2. Summary of RNA-sequencing data

Sample	Raw Reads	Clean reads	Total mapped	Multiple mapped	Uniquely mapped
A8_1	41911084	39852264	29199234 (73.27%)	4266938 (10.71%)	24932296 (62.56%)
A8_2	42811438	40633278	29857610 (73.48%)	4346748 (10.70%)	25510862 (62.78%)
A8_3	46819704	44472148	32691521 (73.51%)	4690867 (10.55%)	28000654 (62.96%)
B8_1	47425206	45066824	33376961 (74.06%)	5141076 (11.41%)	28235885 (62.65%)
B8_2	51087202	48510756	35957118 (74.12%)	5586436 (11.52%)	30370682 (62.61%)
B8_3	47649162	45187490	33588473 (74.33%)	5148132 (11.39%)	28440341 (62.94%)

A8: N0 seeds at 8 HAI. B8: N240 seeds at 8 HAI.

Supplementary Table S3. List of selected genes about protein metabolism that were differentially expressed in B8 VS A8.

Gene ID	Gene Annotation	log2FoldChange	p-value
Traes_5DL_CEBA87E3F	Protein synthesis inhibitor II	2.3508	1.21E-43
Traes_5BL_D6C9D200D1	Protein synthesis inhibitor I	2.1278	9.90E-06
Traes_5DL_DF4EA7BE9	Protein synthesis inhibitor I	1.8587	4.73E-03
Traes_4BL_73E79F141	Beta-amylase	1.4758	6.14E-14
Traes_5BL_BF328BC61	protein phosphatase 2C	0.5886	7.11E-03
Traes_7BS_5ECCA936B	60S ribosomal protein L32-1	0.5176	7.95E-03
Traes_6DL_03A05A5D7	60S ribosomal protein L14-1	0.5014	3.25E-06
Traes_2AL_E513B22A7	Unknown	0.4974	5.87E-04
Traes_1AS_CDFADEA22	40S ribosomal protein	0.4368	8.10E-03
Traes_2BS_9FC5D6F3A	40S ribosomal protein S7	0.3756	2.24E-02
Traes_6AL_FE1447C07	60S ribosomal protein L14-1	0.3373	8.99E-03
Traes_2DS_8318AD213	60S ribosomal protein L36a	0.3308	4.04E-02
Traes_6BL_62CC48C2C	60S ribosomal protein L14-1	0.3308	1.28E-02
Traes_3DL_F1AA506F4	60S ribosomal protein L36-2	0.3281	4.69E-02
Traes_2DL_F311FFC60	60S acidic ribosomal protein P2A	0.3258	1.93E-02
Traes_5AL_3B1FFF708	60S ribosomal protein L34	0.3201	2.39E-02
Traes_1DL_22BFD4845	40S ribosomal protein S15a-1	0.3158	2.71E-02
Traes_6DL_164671C3E	60S ribosomal protein L37-2	0.2995	2.88E-02
Traes_6AL_D5111DA34	60S ribosomal protein L37-3	0.2986	1.62E-02
Traes_4DL_BBA5E7BFC	40S ribosomal protein S21	0.2935	4.28E-02

Supplementary Table S4. Primers used for qRT-PCR.

Primer	Sequence (5' to 3')
Actin-F	CGAAGCGACATACAATTCCATC
Actin-R	GAACCTCCACTGAGAACAACAT
Traes_5DL_CEBA87E3F-F	GTAACGACGCTGCTCCTCTTG
Traes_5DL_CEBA87E3F-R	CTCTTCTTCTCCACCGCCTTG
Traes_5BL_D6C9D200D1-F	TCTGCCGACTATGTCACCTT
Traes_5BL_D6C9D200D1-R	CGATGTCTTGAGCACGATGT
Traes_5BL_E68C461B3-F	CCATTGCTCCAGTTGGCATCTT
Traes_5BL_E68C461B3-R	CCGCTACAACGACCAAACCAT
Traes_2BL_2D440C559-F	CGCCTTCTTCGCTCACTTCAT
Traes_2BL_2D440C559-R	TGTTCGTCTCGTCGCAGTAATC
Traes_3DL_4C255B900-F	AGGACATCATCGGAAGGACTCA
Traes_3DL_4C255B900-R	GCTGGTAGTTCTTGCGTGCTAA
Traes_6BS_89265ECAD-F	TCAGAGCACACGGAGGAGGA
Traes_6BS_89265ECAD-R	CCGAGAAGAGTGGTGAGCAGAT
